# A survey of diet self-efficacy and food intake in students with high and low perceived stress

**DOI:** 10.1186/s12937-015-0026-z

**Published:** 2015-04-23

**Authors:** Robyn S Nastaskin, Alexandra J Fiocco

**Affiliations:** Ryerson University, 350 Victoria Street, M5B 2K3 Toronto, Ontario Canada

**Keywords:** Food intake, Fat, Sodium, Stress, Diet self-efficacy

## Abstract

**Objective:**

Given the rise in obesity and obesity-related disorders, understanding the relationship between stress, self-efficacy and food choice in young adulthood may have implications for preventing negative health outcomes later in life that stem from poor eating habits. The current study examined whether stress levels and diet self-efficacy may be associated with unhealthy eating habits in young adults.

**Methods:**

Male and female undergraduate students (N = 136) completed questionnaires that tap into diet self-efficacy (DSE), perceived stress (PS), sodium, and fat intake. Sex differences in choice of food were predicted, and low levels of perceived stress and high diet self-efficacy were expected to be associated with lower fat and sodium intake.

**Results:**

Findings indicate an interaction between perceived stress and diet self-efficacy on fat intake and a main effect for diet self-efficacy on sodium intake in this population. As expected, low levels of perceived stress and high diet self-efficacy were associated with the lowest levels of fat and sodium intake in students. Findings were driven by females.

**Conclusions:**

This study provides preliminary evidence that diet self-efficacy and perceived stress levels relate to nutrient intake in young adult females, and that increasing diet self-efficacy and reducing perceived stress in young adult females may lead to reductions in fat and sodium intake, leading to healthier eating habits.

## Introduction

The young adult North American population is found to ingest more fat and sodium than is considered healthy. According to Health Canada [1], 25% of males and 23% of females 19 years of age and older report fat intake above the recommended amount (25-35% of total energy intake). In addition, 99% of males and 73% of females aged 19-30 years old reportedly ingest more than the tolerable upper intake level of 2300 mg of sodium per day [1]. The question that arises from these statistics is: what is causing young adults to over-consume fat and sodium?

Over the past few years, reports of increased stress among college and university students have surfaced [2,3]. In a related vein, studies have shown that *stress* is a significant instigator of poor eating behaviors, especially in the young adult population [4]. Animal research shows that stress exposure increases both fat and sodium intake.

The majority of human studies have focused on fat intake in relation to stress. Studies show that females [4-8] and males [9-11] ingest more fat following exposure to a psychosocial stressor. Interestingly, Epel and colleagues [5] found that increased secretion of the stress hormone cortisol following a psychosocial stressor as well as stress induced by completion of visuospatial puzzles and serial subtraction tasks was related to fatty but not sodium-rich foods in female participants.

Fewer studies have examined the relationship between stress and sodium intake. One study by Miller et al. [12] found that males who scored high on hostility and who were more stress-reactive to a psychosocial stressor, reported greater sodium consumption on a food frequency questionnaire and consumed more sodium in the laboratory [12]. The cyclical nature of the stress response and food intake in these subjects demonstrates that sodium intake resulting from stress may only worsen subsequent physiological feelings of stress in individuals by increasing blood pressure [13,14].

Although a number of studies demonstrate an increase in fat intake among highly stressed individuals, a handful of studies have reported decreased intake under stress [15,16]. Epel and colleagues [5] measured dietary restraint (those who are attempting to actively diet and place restraint on their food intake) and serum cortisol resulting from stressors such as visuospatial puzzles, serial subtraction tasks and deliverance of a speech in young adult females. The authors found high cortisol release to be related to intake of fatty but not salty foods, and that overall, psychophysiological responses to stress may induce uhealthy eating [5]. On the contrary, Stone and Brownell (in [15]) examined daily records of stress and eating habits and found that male and females students report eating less when faced with more severe stressors. Mixed findings within the literature are likely related to individual differences; not everyone responds to stress in the same way, and thus not everyone overeats, or eats high-fat or high-sodium foods when stressed. A potential mediating factor to consider in the relationship between stress and food intake is self-efficacy.

Research suggests that self-efficacy affects the cognitive appraisal of a stressor and thus the stress response that ensues [17,18]. General self-efficacy is loosely defined as one’s confidence in his or her ability to manage a demand in the presence of obstacles [19]. Indeed, a number of studies have shown that greater level of general self-efficacy is related to lower reports of stress [18,20-22]. Ebstrup and colleagues [21] examined the role of general self-efficacy in the stress appraisals of male and female participants aged 18-69 years and found that general self-efficacy acts as a buffer to stress by increasing one’s beliefs that he or she is able to overcome the external events or obstacles that are perceived as stressful. Given that self-efficacy decreases perceived stress, it may be suggested that this attribute may moderate the association between stress and nutrient intake. In other words, although stress levels may be heightened in a given situation, greater self-efficacy may reduce one’s tendency to use unhealthy food intake as a way of reducing feelings of stress.

Findings regarding the relationship between self-efficacy and nutrient intake are mixed. Armitage and Connor [23] conducted a study with undergraduate males and females, and found that although self-efficacy did not directly correlate with eating behavior, it was a predictor of intention to reduce fat intake; a potential explanation for this is that their measure of self-efficacy consisted of a non-validated 2-item scale. O’Connor et al. [7] investigated general self-efficacy as a mediator for perceived stress, and also examined the relationship between self-efficacy and health behaviours. O’Connor and colleagues [7] found that stress leads to increased fat consumption; however, greater self-efficacy was associated with lower fat consumption in high stressed men and women. Similar results were found in a study conducted by Royal and Kurtz [24], although their sample consisted only of female undergraduates. In a study by Barrington et al. [25], it was found that high levels of perceived stress are associated with greater fast-food intake, especially among individuals with low eating awareness. However, no associations between self-efficacy and stress or food intake were found [25]. One potential explanation for this negative finding is that fast food intake and self-efficacy were measured using a non-validated 1- and 2-item scale respectively. Fewer studies have assessed the relationship between self-efficacy and sodium intake. A study conducted by Cornelio and colleagues [14] examined behavioral determinants of sodium consumption in individuals with hypertension; the researchers measured self-efficacy using a 3-item scale at baseline and found that higher self-efficacy predicted intention to avoid the use of sodium in cooking and to avoid consumption of foods with high sodium content over a two month period. Women with lower self-efficacy were found to add sodium to foods while cooking, although self-efficacy was not related to actual avoidance of high-sodium foods in either gender [14]; the authors did suggest that interventions to decrease sodium consumption should incorporate changes in self-efficacy.

Mixed findings pertaining to the relationship between stress, self-efficacy and nutrient intake likely result from the use of general self-efficacy scales, which are not specific to food intake behavior. Bandura [17] was one of the most prominent theorists to state that self-efficacy is primarily task-specific, and thus to measure potential behavior outcomes, measures of self-efficacy should be specific to that behavior. Importantly, displaying a high level of general self-efficacy does not indicate that an individual is efficacious in all self-efficacy components [26]. Another possible explanation is that general self-efficacy does not directly map onto eating behaviors and thus diet self-efficacy would be a more appropriate measure in assessing the moderating role of self-efficacy in the relationship between stress and food intake. Diet self-efficacy is one component of self-efficacy that depicts one’s belief in his or her ability to manage a diet even in the face of obstacles such as stress or exposure to unhealthy foods; thus, diet self-efficacy may act as a moderator between perceived stress and food intake behavior.

To date, only one study has assessed the role of diet self-efficacy in the relationship between stress and food intake. Foreyt et al. [27] used a series of questionnaires and found that women reported lower diet self-efficacy and greater levels of stress compared to men. Further, obese participants reported significantly lower diet self-efficacy compared with that of average-weight individuals [27]. Although this study demonstrates positive associations between diet self-efficacy, stress and weight, Foreyt and colleagues did not examine specific intake of nutrients, such as fat and salt, which may influence individuals’ physical health status.

The goal of the current study was to investigate the relationship between perceived stress and fat and salt intake, and to evaluate the moderating role of self-efficacy in young adults. In order to address previous mixed findings on the role of self-efficacy, we measured both general self-efficacy and diet self-efficacy. It was hypothesized that increases in perceived stress would associate with increases in fat and salt intake. It was also hypothesized that diet self-efficacy would moderate the relationship between stress and food intake, in that high stressed individuals with high diet self-efficacy would report lower fat and salt intake compared with high stressed-low diet self-efficacy individuals. Overall, the highest sodium and fat intake was expected in individuals reporting high stress and low diet self-efficacy and the lowest sodium and fat intake was expected in students reporting low stress and high diet self-efficacy; low stress-low diet self-efficacy and high stress-high diet self-efficacy groups were not expected to differ in nutrient intake. Finally, in line with Bandura’s theory, general self-efficacy was not expected to moderate the relationship between perceived stress and nutrient intake.

## Methods

### Ethical considerations

All research conducted within the current study was approved and overlooked by Ryerson University’s Research Ethics Board. Necessary documents, including an ethics proposal, consent and debriefing forms, as well as all questionnaires were submitted and approved prior to conducting the study.

### Measures

In order to test the predicted hypotheses, participants completed six questionnaires, including those that measure levels of perceived stress, self-efficacy, and nutrient intake.

#### Demographics questionnaire

A 15-item demographics questionnaire developed by the primary researcher was used to gain general demographic information from the participants, including age, sex, race, smoking status, program of study, work status, number of exams in the past month, hypertension diagnosis, medications being taken, height, and weight. All items on the demographics questionnaire were self-reported, with the exception of participants’ height and weight, which was measured by researcher to calculate Body Mass Index (BMI).

#### Eating habits confidence scale

A 20-item self-report diet self-efficacy questionnaire designed to evaluate an individual’s belief in his or her ability to successfully avoid eating certain unhealthy foods, namely high-fat and high-sodium foods. Participants’ final scores on this scale may range from 0-100. An example of an item in the EHCS is “How sure are you that you can stick to your low fat, low salt foods when there is high fat, high salt food readily available at a party?”. This questionnaire has been validated in the target population for the current study [28], and deemed a reliable measure of diet self-efficacy (alpha = 0.9) [29].

#### General perceived self-efficacy scale

A 10-item self-report measure used to assess general self-efficacy. The questionnaire is designed to measure one’s general sense of perceived self-efficacy, with potential scores ranging from 0-40. An example of an item in the GPSES is “It is easy for me to stick to my aims and accomplish my goals”. This questionnaire has been validated in the target population for this study [30], and has been deemed a reliable measure, with Cronbach’s alpha ranging from 0.8-0.9 [31,32].

#### Cohen’s perceived stress scale

This scale is a 14-item self-report questionnaire with a maximum potential score of 56. This scale is commonly used to assess an individual’s perception of stress over a 1-month period. An example of an item in this questionnaire is “In the past month, how often have you felt nervous or stressed?”. This scale has been deemed reliable and has been validated in the target population for this study [33]. This scale has a measured reliability of Cronbach’s alpha of 0.9 [34,35].

#### Block fat screener

A 17-item self-report questionnaire used to evaluate an individual’s fat intake over a 1-month period. This screener evaluates how often an individual has eaten a certain food in the past month. An example of an item found on this screener is “How often have you eaten doughnuts, pastries, cake, cookies (not low-fat) within the past month?”. This screener has been validated in the target population for this study [36], and has been deemed reliable, with a Cronbach’s alpha between 0.7 and 0.9 [37].

#### Block sodium screener

A 28-item self-report questionnaire used to evaluate an individual’s sodium intake over a 1-month period. This screener evaluates how often an individual has eaten specific types of food in the past month, and how often they have done so within the average day. An example of an item found on this screener is “How often have you eaten salad dressing in the past month, and within a day?”. The Block food screeners have been deemed validated in the target population for studies similar to the current, although a reliability study has yet to be conducted [36].

### Participant characteristics

Undergraduate students (n = 136; 23 male, 113 female; M_age_ = 20.62, SD = 3.41) were recruited from Ryerson University in Toronto, Ontario, Canada. Participants were eligible on the condition that they were enrolled in the Introductory Psychology courses at Ryerson University. Thus, students who were completing any degree which would allow them to take the Introductory Psychology courses were eligible to participate in the study. Those who were eligible were recruited through Sona, the university’s online participant pool, which was then made up of hundreds of students. Fifty-five percent of the sample population identified themselves as Caucasian and the average body mass index (BMI) within the sample was 21.93 (SD = 4.97).

### Procedure

Upon arrival at the Stress and Healthy Aging Research Lab at Ryerson University, participants were asked to read and sign an informed consent form. Following consent, participants completed the Eating Habits Confidence Scale, the General Perceived Self-Efficacy Scale, the Perceived Stress Scale, the Block Fat Screener, the Block Sodium Screener, and the demographics questionnaire. Following completion of the questionnaire battery, the primary researcher weighed the participant and measured the individual’s height in order to calculate BMI. Upon completion of the study, participants were debriefed.

### Research design and statistical analyses

Using a survey-based cross-sectional design, all participants completed the questionnaires in the same order in order to reduce contingency biases in responses to subsequent questionnaires.

Variables of perceived stress (PS), diet self-efficacy (DSE), general self-efficacy, and fat and sodium intake were assessed for normality and outliers. All variables were normally distributed and 6 outliers were removed from the data due to age and health conditions. One male and two female participants were removed from the dataset based on their age and program; they were all significantly older than the mean age (38, 40 and 58 years of age) in the continuing education program and they reported health concerns that may impact nutrient intake (hypertension and prescribed lowered sodium consumption). Two males were removed from the data set due to their outlying age (41 and 48 years of age) and health conditions (high cholesterol and Brown Adipose Tissue). One male was removed due to his stress score being significantly above the mean (more than three standard deviations). Following removal of these 6 participants, a total of 130 participants (19 male, 111 female) were included in subsequent analyses.

To assess the association between PS, DSE and fat and sodium intake, separate linear regression analyses were performed. In each model, PS, DSE and their interaction (PS × DSE) were entered in step one, followed by the covariates age, race, and sex in step two. This model was conducted for both fat and salt intake as the outcome variable of interest. Further, the same analyses were conducted using general self-efficacy (GSE) to confirm that diet self-efficacy is a more relevant measure than general self-efficacy in assessing the moderating role between stress and fat and sodium intake.

In order to assess and visualize the interaction between stress and self-efficacy on fat and sodium intake, a median split was created for perceived stress (HighPS-LowPS) and diet self-efficacy (HighDSE-LowDSE), which then enabled the creation of four groups: highPS-highDSE (n = 31), lowPS-lowDSE (n = 30), highPS-lowDSE (n = 36), and lowPS-highDSE (n = 33). Analyses of variances followed by post-hoc group comparisons were performed to assess group differences in the combined effects of PS and DSE on sodium and fat intake. Given that the proposed hypotheses were based on group (e.g. high PS and low DSE is associated with greater sodium intake), ANOVAs were conducted irrespective of the aforementioned regression analyses.

Finally, subgroup analyses were conducted to explore the relationship between stress and self-efficacy on fat and sodium intake in female and male participants. All analyses were performed using SPSS (Statistical Package for the Social Sciences) and results were considered significant at the 5% alpha level.

## Results

For the purpose of the current analyses, *d* is meant to indicate Cohen’s Effect Size. A larger effect size indicates that the difference between means is significant [38]. In addition, for the purpose of these analyses, the term ‘intake’ is meant to indicate fat and sodium scores based on self-report food intake questionnaires. All additional calculations with respect to fat and sodium intake (e.g. mg/day) are based on intake scores.

### Sample characteristics

Participants were on average 20.62 years of age (SD = 3.41), with 85% of the sample being female. Fifty-five percent of the sample identified themselves as Caucasian, and the average BMI of the sample was 21.93 (SD = 4.97). See Table 1.

**Table 1:** **Sample characteristics**

Variable	Mean (SD) or %
**Demographics**	
Age (Min = 18.0, Max = 38.0)	20.62 (3.41)
Sex (% female)	85
Race (% Caucasian/White)	55
BMI (Min = 14.87, Max = 35.2)	21.93 (4.97)
Smoking status (% yes)	6.9
**Program**	
Psychology	19
Social work	17
Biology	12
Nursing	10
Other	42
**Work**	
No (%)	44
Part time (%)	55
Full time (%)	1
**Exams**	
0 (%)	6
1 to 2 (%)	12
3 to 4 (%)	25
5 to 6 (%)	33
7+ (%)	24
Hypertension (%yes)	1
Medication (%yes)	18
**Stress and self efficacy**	
PS (Min = 7.0, Max = 40.0)	26.72 (7.43)
GSE (Min = 20.0, Max = 40.0)	30.31 (4.0)
DSE (Min = 25.0, Max = 100)	75.88 (12.73)
**Dietary intake**	
Sodium score (Min = 8.0, Max = 64.00)	30.21 (10.85)
Sodium (mg/day) (Min = 194.6, Max = 6512.5)	2983.7 (1184.16)
Fat score (Min = 4.0, Max = 48.0)	20.66 (7.96)
Percent fat (%) (Min = 24.0, Max = 50.9)	34.16 (4.82)
Saturated fat (g) (Min = 9.4, Max = 48.1)	24.57 (7.16)
Total fat (g) (Min = 49.5, Max = 159.1)	91.94 (19.31)
Cholesterol (mg) (Min = 96.55, Max = 476.35)	254.06 (69.99)

Average GSE, DSE and PS scores were also calculated. The average GSE rating was 30.31 (SD = 3.96). The average DSE rating was 75.88 (SD = 12.73) and the average PS rating was 26.72 (SD = 7.43). See Table 1 for additional calculated means.

### Sample characteristics by sex

Males and female participants differed in their report of GSE, DSE, and PS. Males reported significantly higher levels of GSE (F(1,128) = 5.91, *p* = 0.02; *d =* .67) than females, and females reported higher levels of PS (F(1,128) = 7.06, *p =* 0.01; *d* = .77) than males. There were no significant differences between males and females in terms of reported DSE (F(1,128) = 0.06, *p =* 0.81; *d* = .07).

Males and females differed in their reports of both fat and sodium intake. Saturated fat (F(1,128) = 6.96, *p =* 0.01; *d* = .66), cholesterol (F(1,128) = 15.69, *p* < 0.001; *d* = .98), and sodium intake in mg per day (F(1,128) = 6.89, *p* = 0.01; *d* = .68) was found to be significantly greater in males compared with female intake scores.

### Effects of stress and diet self-efficacy on reported fat intake

Unadjusted regression analyses showed an interaction effect between PS and DSE (ß = -1.07, *p* = 0.04) on fat score. These findings did not change when controlling for age, sex, and race.

**Figure 1:**
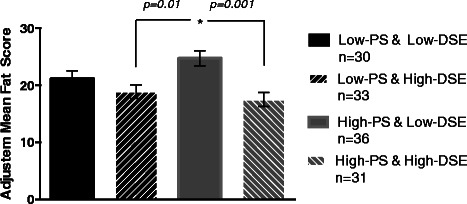
Adjusted mean fat intake score (and standard errors) based on high and low PS and DSE.

Mirroring the aforementioned regression analyses, ANOVA revealed a significant PSxDSE interaction effect on reported fat intake (F(3,125) = 5.83, *p =* 0.001; *r* = .35). Controlling for age, sex and race, ANCOVA revealed the same result, suggesting a significant PS×DSE effect on reported fat intake (F(3,123) = 5.36, *p* = 0.002; *partial ƞ*^*2*^ = .12). Subsequent post-hoc Tukey tests revealed that reported fat intake of highPS-lowDSE participants significantly differed from highPS-highDSE participants (*p* = 0.001) and from lowPS-highDSE participants (*p* = 0.01), but not from lowPS-lowDSE participants (*p =* 0.23) (see Table 2 for adjusted means). More specifically, highPS-lowDSE participants demonstrated significantly greater levels of reported fat intake than highPS-lowDSE participants and lowPS-highDSE participants. See Figure 1.

**Table 2:** **Adjusted means from DSExPS interactions for fat score**

Group	Adjusted mean	Standard error
LowDSExLowPS	20.89	1.41
LowDSExHighPS	24.78	1.30
HighDSExLowPS	18.80	1.33
HighDSExHighPS	17.88	1.37

### Effects of stress and diet self-efficacy on reported sodium intake

Unadjusted regression analyses revealed no interaction effect between DSE and PS. A main effect was revealed for DSE (ß = -0.33, *p* < 0.001) with trending effects for PS (ß = 0.15, *p =* 0.07) on sodium score. Specifically, participants who reported higher levels of DSE also reported lower levels of sodium intake, independent of perceived stress. These findings did not change when controlling for age, sex, and race.

**Figure 2:**
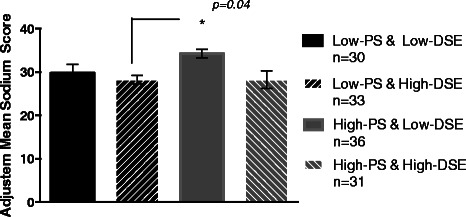
Adjusted mean sodium intake score (and standard errors) based on high and low PS and DSE.

ANOVA revealed a significant PSxDSE interaction effect on sodium intake at the trend level (F(3,126) = 2.47, *p =* 0.07; *r* = .23). A similar interaction effect was found after controlling for age, sex, and race (F(3, 123) = 2.31, *p* = 0.09; *partial ƞ*^*2*^ = .05). Although the interaction was not statistically significant, subsequent post-hoc comparisons were conducted (see Table 3 for adjusted means) which revealed that reported sodium intake was significantly higher in highPS-lowDSE participants compared with lowPS-highDSE participants (*p* = 0.04); although not statistically significant, sodium intake in highPS-lowDSE participants was also greater compared with highPS-highDSE participants (*p =* 0.07) and lowPS-lowDSE participants (*p =* 0.36). See Figure 2.

**Table 3:** **Adjusted means from DSExPS interactions for sodium score**

Group	Adjusted mean	Standard error
LowDSExLowPS	29.49	2.00
LowDSExHighPS	34.29	1.84
HighDSExLowPS	28.13	1.89
HighDSExHighPS	28.55	1.94

### General self-efficacy as a moderator for fat and sodium intake

Regression analyses revealed no significant associations between GSE and fat intake (ß = 0.12, *p =* 0.25), or interaction effects of PS and GSE on fat intake (ß = -0.62, *p =* 0.26). Regression analyses also revealed no significant associations between GSE and sodium intake (ß = -0.31, *p* = 0.31), or interactions between PS and GSE on sodium intake (ß = -0.44, *p* = 0.42).

### Additional variables considered

Additional variables such as BMI, work and school commitments, age, race, and medications being taken were measured. None of these variables were found to correlate with nutrient intake, PS or DSE. Regression analyses and ANOVAs revealed no significant effects of an association between BMI, and PS or DSE.

## Discussion

A robust association exists between stress and increased fat and sodium intake. In light of the growing obesity epidemic and an increase in the amount of stress being reported among college and university students, the current study evaluated perceived stress, self-efficacy and sodium and fat intake in a group of undergraduate students.

Overall, reported perceived stress within this sample was slightly above the standardized norm [39]. Current findings also coincide with previous research suggesting that unhealthy food intake is a common coping mechanism implemented in response to stress in undergraduate students [4].

Previous studies have demonstrated an increase in food intake as a result of increases in reported stress [5,40], but have failed to focus on the specific nutrients that high-stressed individuals tend to gear towards, such as foods that are high in sodium and fat. The current study has refined this association by examining fat and sodium intake instead of using more general measures of food intake. However, based on the current results, stress alone does not contribute to nutrient intake; rather, the effects of stress on sodium and fat intake are dependent on an individual’s level of diet-self efficacy. The combination of high stress and low diet self-efficacy appears to be associated with the greatest amount of reported fat and sodium intake, and the combination of low levels of stress paired with high diet self-efficacy seems to be associated with the lowest reported intake of these nutrients. However, it should be noted that not all post-hoc comparisons were statistically significant; thus these findings must be interpreted with caution.

Research suggests that stress-induced eating of fatty and high-sodium foods may be a contributing factor to the development of obesity - resulting from energy intake exceeding energy output over a long period of time. These diet patterns are also shown to be associated with a number of health problems [41-47]).

Previous studies have either examined general self-efficacy or general food intake (e.g. caloric intake), which have led to mixed findings. The current study shows the importance of matching self-efficacy to the type of behavior under investigation. Indeed, according to Bandura’s [26] theory, it is important to focus on domain-specific self-efficacy, as general self-efficacy does not appear to consistently play a role in specific health behaviors. The present findings support this premise as general self-efficacy was not associated with nutrient intake, nor did it moderate the relationship between stress and nutrient intake. Specifically, this study suggests that the interaction between perceived stress and diet self-efficacy plays an integral role in determining sodium and fat intake. The distinctive moderating effect of diet self-efficacy compared to general self-efficacy reported in this study may explain the mixed and inconclusive findings within the literature. This distinction should also be considered in future research that examines other outcomes, such as assessing the moderating effect of exercise self-efficacy on physical activity behavior.

Interestingly, body mass index was not associated with fat and sodium intake. This may be due to other life-style behaviors that were not investigated (such as exercise), which may moderate the relationship between BMI, fat and sodium. Additionally, stress-inducing situations (e.g. number of exams and assignments one was recently required to complete) were not associated with perceived stress. This lack of association may be related to the importance of “perception” – as previously mentioned, one’s stress level is highly dependent on perception of the stressors that are present in one’s life [48]. The perception of stress is relative and involves other external factors such as interpersonal stressors or health stressors that may contribute to overall stress scores; five exams for one student may be considered as stressful as one exam for another student.

Although the present findings are important and significantly contribute to the existing literature, this study was not without limitations. First, the small number of male participants did not provide enough power to assess sex differences in the relationship between stress, diet self-efficacy and nutrient intake. However, based on observation of the means, male undergraduate students seem to be demonstrating the same pattern outcomes as females (data not shown). Second, the sample was relatively homogeneous, consisting of young adults (Age range = 18-38 years) living primarily in the Greater Toronto Area, which may undermine generalizability of the findings. Finally, the use of self-report questionnaires may be subject to recall bias and self-report bias that may have overestimated or underestimated the results. Future research should examine fat and sodium intake using a larger sample of male participants in order to perform subgroup analyses and investigate sex differences. Sugar intake should also be considered, as it is well-known that high sugar intake can put individuals at risk for negative health outcomes such as diabetes.

As young adults continue to ingest large amounts of sodium and fat, their risk for health conditions such as obesity, hypertension, cardiovascular disease and cognitive impairment also rise. The findings of the current study have important implications for the prevention of the aforementioned detrimental health conditions. Finally, these findings provide insight into the theoretical notion that improvements in diet self-efficacy (but not general self-efficacy), and reductions in perceived stress levels may reduce fat and sodium intake, thus reducing young adults’ risk of developing poor health conditions.
